# Supplementary material for: Comparative effectiveness of ultrasound-guided and anatomic landmark percutaneous dilatational tracheostomy: A systematic review and meta-analysis
Source: PLoS One. 2021 Oct 28;16(10):e0258972. doi: 10.1371/journal.pone.0258972 (PMC8553067; doi:10.1371/journal.pone.0258972)
Supplement: Attachment — Submitted filename: Response to Reviewers0808.docx [file pone.0258972.s003.docx]

**Dear Editor:**
 Enclosed is our revised manuscript (Re: PONE-D-21-11232 Comparative effectiveness of ultrasound-guided and anatomic landmark percutaneous dilatational tracheostomy: A systematic review and meta-analysis). We appreciate your constructive comments, and we have made revisions accordingly.

Please find our responses to your comments below.

**1. The authors have done good job in their manuscript, as reflected in the reviewers' comments. I agree that for this paper to be published, a thorough English editing is needed, by a professional English Editor**

Answer: We appreciate your comments. This manuscript has been revised by a professional English editing service (https://www.aje.com).

**Dear Reviewer #1 :**

Enclosed is our revised manuscript (Re: PONE-D-21-11232 Comparative effectiveness of ultrasound-guided and anatomic landmark percutaneous dilatational tracheostomy: a systematic review and meta-analysis). We appreciate your constructive comments, and we have made revisions accordingly.

Please find our responses to your comments below.

Reviewer #1

**1. Safety of BPT - in the introduction there is some concern regarding high ICP and high AW pressure with BPT. the BPT safety has been reported in other trials. regarding the high ICP- bronchoscopy is done in TBI patients for other indications as well and it is not clear from the reference if that is a consequence of insufficient sedation. as for high AW pressures - again- the high AW pressure in BPT is secondary to high resistance and so the relation to PNX is loose.**

Answer: We appreciate your comments. We added reference No. 6 to explain that the benefit of decreasing complications was controversial under BGT in the “Introduction” section. The previous sentence that BGT was not suitable for traumatic brain injury patients has been removed. We also edited the explanation for BGT-induced high airway pressure and removed the original pneumothorax complications in the revised manuscript.

On page 4 line 9: BGT provides benefits in the real-time confirmation of needle placement, midline positioning of the needle, and avoidance of posterior tracheal wall injury [4,5]. However, the benefit of reducing the complication rate is not significantly observed [6,7]. A previous study also noted that increased airway resistance during BGT would secondary increase high airway pressure [8]. Therefore, considering patient safety and cost effectiveness, BGT is challenging among PDT procedures [7].

**2. Total tracheostomy placement time is considered in only 2 papers (the third was measured differently) and so the data are limited to conclude that UPT is not associated with time reduction, or the other way around.**

Answer: We agree with your comments. Considering that the total tracheostomy placement time was considered in only two papers, we modified the conclusion that the comparison of total tracheostomy placement time between UGT and LT should be further investigated.

On Page 3 line 3: The total tracheostomy placement time comparison between UGI and LT was inconclusive.

On Page 23 line 1: Since the total tracheostomy placement time was considered in only two articles, it was difficult to conclude whether UGT could reduce total tracheostomy placement time compared with LT.

On Page 24 line 6: The total tracheostomy placement time between UGT and LT should be further investigated.

**Dear Reviewer #2:**

Enclosed is our revised manuscript (Re: PONE-D-21-11232 Comparative effectiveness of ultrasound-guided and anatomic landmark percutaneous dilatational tracheostomy: a systematic review and meta-analysis). We appreciate your constructive comments, and we have made revisions accordingly.

Please find our responses to your comments below.

**1.-While the English in the study is intelligible, the manuscript, and especially the discussion section would benefit from linguistic editing.**

Answer: We appreciate your comments. We have revised the manuscript, especially the “Discussion” section, for coherence. Additionally, this manuscript has been revised by a professional English editing service (https://www.aje.com).

**2.- Abstract: Numbered references are not used in abstracts. Please revise.
- Results: The font in figure 5 is too small, and it is lacking headlines.**

Answer: We appreciated your suggestions. The numbered references were deleted from the “Abstract,” and Figure 5 was modified for clarity in the revised manuscript. Please see the Abstract and Figure 5.
